# Iris metastasis of gastric adenocarcinoma

**DOI:** 10.1186/s12957-016-0840-6

**Published:** 2016-03-08

**Authors:** Ali Riza Cenk Celebi, Ayse Ebru Kilavuzoglu, U. Emrah Altiparmak, C. Banu Cosar, Abdullah Ozkiris

**Affiliations:** Acibadem University School of Medicine, Istanbul, Turkey

**Keywords:** Gastric adenocarcinoma, Iris, Masquerade, Metastasis, Uveitis

## Abstract

**Background:**

Iris metastasis in patients with gastric cancer is extremely rare. Herein, it is aimed to report on a patient with gastric adenocarcinoma and iris metastasis.

**Case presentation:**

A 65-year-old patient with the history of gastric cancer was admitted for eye pain and eye redness on his left eye. There was ciliary injection, severe +4 cells with hypopyon in the anterior chamber and a solitary, friable, yellow-white, fleshy-creamy vascularized 2 mm × 4 mm mass on the upper nasal part of the iris within the left eye. The presented patient’s mass lesion in the iris fulfilled the criteria of the metastatic iris lesion’s appearance. The ocular metastasis occurred during chemotherapy.

**Conclusions:**

Iris metastasis can masquerade as iridocyclitis with pseudohypopyon or glaucoma. In patients with a history of gastric cancer that present with an iris mass, uveitis, and high intraocular pressure, ocular metastasis of gastric cancer should be a consideration.

## Background

Metastasis from systemic carcinoma to the eye was reported to occur in 4 % [1] and 8 % [2] of cases in two autopsy-based studies. The choroid is the most frequent site of ocular metastasis of systemic cancer. The metastatic spread to the iris was uncommon and quite rare [3].

Gastric adenocarcinoma is among the most common causes of cancer-related mortality worldwide [4]. Gastric adenocarcinoma usually develops slowly, but eventually progresses to multiple sites of metastasis [5]. Choroidal metastatic tumors of gastric cancer origin—though rare—have been reported [5–7]. A recent study that included 160 metastatic tumors of systemic cancer origin in the iris in 107 eyes of 104 patients reported no cases of metastasis in the iris of gastric cancer origin [8].The iris is an extremely rare site of gastric cancer metastasis. To the best of our knowledge, the English-language literature includes just one such case, which was reported by Imamura et al. [9]. In that case, metastasis in the iris occurred 3 years after gastric cancer was diagnosed. Herein, we report on the second case of gastric adenocarcinoma metastasizing to the iris; metastasis occurred 8 months after the cancer was diagnosed and while the patient was receiving systemic chemotherapy.

## Case presentation

A 65-year-old male presented in April 2015 with a chief complaint of redness and pain in the left eye within the last 2 weeks. Past medical history included highly dysplastic undifferentiated gastric adenocarcinoma that was diagnosed by gastric biopsy via esophagogastroduodenoscopy in August 2014. The patient had undergone 18F-fluorodeoxyglucose-positron emission tomography/computed tomography (18F-FDG-PET/CT), which showed multiple uptakes in the distal stomach at the antrum and pylorus, lesser curvature, peripancreatic lymph nodes, and at multiple sites in the liver, but no uptake by the globe or periocular region. An additional biopsy specimen was obtained from a mass in the liver in September 2014, which showed metastasis of gastric adenocarcinoma. To differentiate between primary and a metastatic liver mass lesion, immunohistochemistry was performed. The liver lesion from gastrointestinal system was considered metastatic with the strong cytokeratin positivity, strong CDX2 focal positivity, focal cytokeratin 20 positivity, and strong cytokeratin 7 positivity. Both the primary lesion from the gastric biopsy and the metastatic liver lesion shared the same high-grade dysplastic gland formation under microscopy. The patient’s final diagnosis was stage 4 gastric cancer with multiple metastases.

**Fig. 1:**
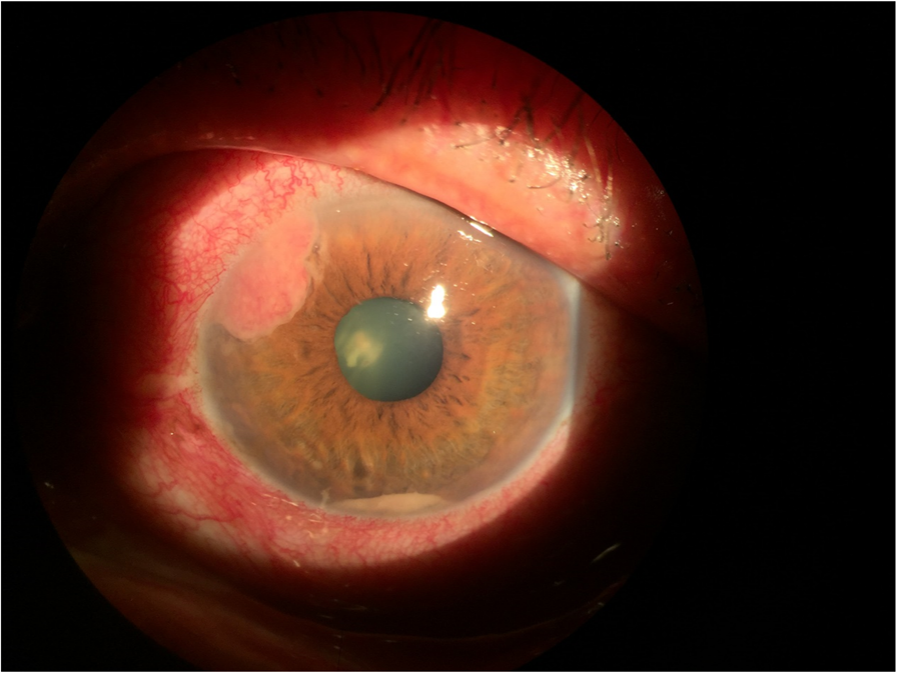
The patient’s left eye. Solitary, friable, yellow-white, fleshy, creamy vascularized 2 mm × 4 mm mass on the nasal upper part of the iris and a small 0.5 mm × 0.5 mm yellow-white creamy mass at the 7 o’clock position on the mid-peripheral iris

Chemotherapy was initiated in September 2014 with docetaxel, cisplatin, leucovorin, and 5-FU (modified DCF protocol). The modified DCF protocol was applied as follows: (1) 40 mg/m^2^ in total 75 mg docetaxel with 500 cc 5 % dextrose in 1 h intravenous infusion on the first day, (2) 40 mg/m^2^ in total 75 mg cisplatin with 500 cc isotonic saline in 1 h intravenous infusion, (3) 400 mg/m^2^ in total 770 mg leucovorin in 500 cc 5 % dextrose in 1 h intravenous infusion, and (4) 400 mg/m^2^ in total 770 mg 5-FU intravenous bolus injection. On the first day following intravenous bolus injection, 5-FU was administered 2000 mg/m^2^ in total 3860 mg with infusion pump continuously during the first and second day. This treatment scheme was repeated 2 weeks apart. Follow-up 18F-FDG-PET/CT in January 2015 showed stable gastric lesions and no uptake within the globe itself. The patient’s visual complaints started in April 2015. Visual acuity was 20/20 in the right eye (*oculus dexter* (OD)) and 20/50 in the left eye (*oculus sinister* (OS)). Intraocular pressure was 16 mmHg OD and 32 mmHg OS. There was ciliary injection, minimal upper nasally located corneal edema, severe +4 cells with hypopyon in the anterior chamber and a solitary, friable, yellow-white, fleshy-creamy vascularized 2 mm × 4 mm mass on the upper nasal part of the iris within OS (Fig. 1). During gonioscopy examination, the mass obscured the angle structures in the upper nasal portion of the iris. In addition, there was a 0.5 mm × 0.5 mm yellow-white creamy mass in the 7 o’clock position on the mid-peripheral iris (Fig. 1). Fundus examination was normal OS. The right eye was pseudophakic, and the ophthalmic examination was otherwise unremarkable.

Two days after identifying the possible iris metastasis, orbital MRI was performed to define the posterior extension of the mass before the needle aspiration biopsy; the mass was shown to be limited to the iris; no other mass lesions were noted at the ciliary body, choroid, optic nerve, extraocular muscles, or intra-orbital fat. Fine-needle aspiration biopsy was scheduled 1 day after the report of orbital MRI was obtained, but the patient passed away after a sudden respiratory arrest, nearly 10 months after the primary tumor was diagnosed.

### Discussion

Between 90 and 95 % cases of ocular metastasis from systemic cancer occur in the choroid [3]. A survey of 950 metastatic foci in 520 eyes with uvea metastasis showed involvement of the choroid in 88 % and iris in 9 % of the eyes [3]. It was posited that uvea metastasis occurs in the choroid more commonly than in the iris because of the rich blood supply to the posterior choroid via the posterior ciliary arteries. This facilitates a greater volume of blood flow and is associated with a greater risk of metastatic emboli, as compared to the anterior ciliary vessels that supply blood to the iris [10].

Gastric adenocarcinoma metastasizes to numerous sites; the most common sites are the liver and non-regional or distant lymph nodes, whereas choroidal and iris metastases are very rare [5]. To the best of our knowledge in the English-language literature, only one similar case of isolated iris metastasis of gastric cancer has been reported [9], and the presented case is the second. In the earlier case, the iris mass was white and lacework-like and encompassed the entire area of the iris. In contrast, the iris mass was solitary, friable, yellow-white, and fleshy and presented with a pronounced ciliary injection, in the current case.

A recent study about iris metastasis of systemic cancer among 104 patients reported that only three patients had iris metastasis of esophageal cancer, and only two patients had iris metastasis of colon cancer; no patient had iris metastasis of gastric cancer [8]. One case report describes a patient with iris metastasis from esophagus as the primary site of the gastrointestinal tract system [11]. In that case, iris metastasis was controlled using systemic chemotherapy. Unfortunately, the iris metastasis in the present case was diagnosed while the patient was undergoing systemic chemotherapy. Shields et al. [3] studied 950 uvea metastases in 520 eyes and reported that gastrointestinal tract carcinoma was the primary site in 4 % of the uvea metastatic tumors. Soysal et al. [12] reported one uvea metastasis from the gastrointestinal tract in a survey of 38 eyes, in which the choroid was the only site of metastasis of gastrointestinal carcinoma. Gastric carcinomas generally prefer metastasizing to the choroid [6]; however, multiple cases of gastric adenocarcinoma metastases to the eyelid and conjunctiva [13] and optic nerve [14] have been reported versus just one case report of isolated iris metastasis of gastric cancer [9].

Iris metastasis can manifest with a stromal nodule, ill-defined iris thickening, pain, iridocyclitis, or hyphema. In 1995, Shields et al. [8] published a clinically based analysis of 40 cases of iris metastasis during a 20-year period, reporting that the patients’ primary complaints were blurred vision, ocular pain, and eye redness. Ocular pain was presumably related to secondary glaucoma, iridocyclitis, or scleral invasion of the tumor. Ocular pain in the presented case was thought to be associated with secondary glaucoma and iridocyclitis. Another study by Shields et al. [15] reported that visual acuity was 20/40 or better in 68 % of cases with iris metastasis of systemic cancer origin, but unfortunately, over 90 % of the patients with visual acuity less than 20/40 had ipsilateral choroidal metastasis with retinal detachment. The presented patient underwent orbital MRI to determine if he had ipsilateral metastasis, but choroidal infiltration was not observed. In the most recent study by Shields et al. [8], it was reported that the most common presenting complaint in patients with iris metastasis (32 %) was ocular pain.

Pseudohypopyon as a clinical feature of metastatic tumors in the iris was observed in only nine cases in a study [8]; this rare entity was also noted in the presented case. In that earlier study [8], fine-needle aspiration biopsy was performed for cytological confirmation. In unavailable cases for fine needle aspiration biopsy, the appearance of the mass lesion yields significant importance of understanding the metastasis. Metastatic mass lesions generally appear in a yellow nodular formation with a mean diameter of 5.9 mm. Although there is considerable variability, an earlier study reported that the characteristic features of an iris metastasis can be summarized as a solitary, friable, yellow-white or fleshy mass that frequently releases tumor cells into the anterior chamber, which can stimulate an inflammatory process and lead to secondary glaucoma [15]. The presented patient’s mass lesion in the iris fulfilled these criteria, and metastatic iris tumor was the definitive diagnosis. According to Shields et al. (who have +40 years of experience in ocular oncology and published the two largest studies [8, 15] on iris metastasis from systemic cancers), the best method for diagnosing iris metastasis is observation of the typical tumor via slit-lamp biomicroscopy in a patient with a history of malignancy. When the diagnosis is uncertain, the best ancillary diagnostic method is fine needle aspiration biopsy of the suspicious iris mass [15]. However, we were unable to perform fine needle aspiration biopsy since the patient passed away. The major limitation in this case report is that we do not have the pathological confirmation of the iris metastasis. However, we would like to impress in this case study that one could come up with the diagnosis of iris metastasis from gastric cancer with the appearance of the multiple iris mass lesions in a patient with the history of gastric cancer, showing the specific features of the iris mass lesions as described before in patients with the history of systemic cancer [8]. Metastatic iris lesions were reported to present as isolated lesions, without concurrent choroid and/or ciliary body involvement in 61 % of cases [8], as in the presented case.

The treatment method for iris metastasis depends on a number of factors. If a patient has advanced, widespread systemic metastasis and the affected eye is relatively asymptomatic, no immediate treatment for the iris metastasis is necessary. Many patients with iris metastasis benefit from concurrent chemotherapy, in terms of local tumor control [15]; however, when chemotherapy does not result in adequate local tumor control, external beam irradiation is considered [8, 15]. Ocular outcome in patients with iris metastasis was reported to be positive, with tumor control in 95 % of cases [8]; however, the overall systemic prognosis was generally poor [8]. Adequate follow-up was reported in 98 of 104 patients; 85 of the patients died within a median period of 10 months after diagnosis of iris metastasis [8], whereas the presented patient died just 1 month after iris metastasis was diagnosed.

## Conclusions

Iris metastasis of a primary tumor originating from the gastric region is rare. The presented case shows that gastric adenocarcinoma can cause isolated metastasis in the iris. The presented case was diagnosed based on clinical history and ocular examination. Iris metastasis can masquerade as iridocyclitis with pseudohypopyon or glaucoma. In patients with a history of gastric cancer that presents with an iris mass, uveitis, and high IOP, ocular metastasis of gastric cancer should be considered.

## Consent

Written informed consent was obtained from the parents of the deceased patient for publication of this case report and any accompanying images. A copy of the written consent is available for review by the Editor-in-Chief of this journal.

## References

[1] Bloch RS, Gartner S (1971). The incidence of ocular metastatic carcinoma. Arch Ophthalmol.

[2] Nelson CC, Hertzberg BS, Klintworth GK (1983). A histopathologic study of 716 unselected eyes in patients with cancer at the time of death. Am J Ophthalmol.

[3] Shields CL, Shields JA, Gross NE, Schwartz GP, Lally SE (1997). Survey of 520 eyes with uveal metastases. Ophthalmology.

[4] Jemal A, Bray F, Center MM, Ferlay J, Ward E, Forman D (2001). CA Cancer J Clin.

[5] Kawai S, Nishida T, Hayashi Y, Ezaki H, Yamada T, Shinzaki S (2013). Choroidal and cutaneous metastasis from gastric adenocarcinoma. World J Gastroenterol.

[6] Sahin A, Kiratli H (2007). Choroidal metastasis as a first sign of recurrence in a patient with gastric adenocarcinoma. Can J Ophthalmol.

[7] Liszauer AD, Wiens JJ, Brownstein S, Deschênes J (1991). Gastric linitis plastica metastatic to the uvea. Can J Ophthalmol.

[8] Shields CL, Kaliki S, Crabtree GS, Peshtani A, Morton S, Anand RA (2015). Iris metastasis from systemic cancer in 104 patients: the 2014 Jerry A. Shields Lecture. Cornea.

[9] Imamura Y, Suzuki M, Nakajima KI, Murata H (2001). Gastric signet ring cell adenocarcinoma metastatic to the iris. Am J Ophthalmol.

[10] Ferry AP, Font RL (1974). Carcinoma metastatic to the eye and orbit. I. A clinicopathologic study of 227 cases. Arch Ophthalmol.

[11] Ichiki Y, Morita M, Yano K, Sugio K, Yasumoto K, Hirose N (2005). Iris metastasis of esophageal cancer. Ann Thorac Surg.

[12] Soysal HG (2007). Metastatic tumors of the uvea in 38 eyes. Can J Ophthalmol.

[13] Küchle M, Holbach L, Schlötzer-Schrehardt U (1992). Gastric adenocarcinoma presenting as an eyelid and conjunctival mass. Eur J Ophthalmol.

[14] Sung JU, Lam BL, Curtin VT, Tse DT (1998). Metastatic gastric carcinoma to the optic nerve. Arch Ophthalmol.

[15] Shields JA, Shields CL, Kiratli H, de Potter P (1995). Metastatic tumors to the iris in 40 patients. Am J Ophthalmol.

